# Tablet computers with mobile electronic medical records enhance clinical routine and promote bedside time: a controlled prospective crossover study

**DOI:** 10.1007/s00415-014-7581-7

**Published:** 2014-12-05

**Authors:** Robert Fleischmann, Julian Duhm, Hagen Hupperts, Stephan A. Brandt

**Affiliations:** 1 Department of Neurology, Charité-Universitätsmedizin Berlin, Charitéplatz 1, 10117 Berlin, Germany; 2 Department of Medical Information Technology, Charité-Universitätsmedizin Berlin, Charitéplatz 1, 10117 Berlin, Germany

**Keywords:** Tablet PC, iPad, Ward round, Efficiency, Clinical routine, Electronic medical record

## Abstract

Demographic changes require physicians to deliver needed services with fewer resources. Neurology as an interdisciplinary domain involves complex diagnostic procedures and time-consuming data handling. Tablet PCs might streamline clinical workflow through mobile access to patient data. This study examined the impact of tablets running an electronic medical record on ward round performance. We hypothesised that tablet use should reduce ward round time and decrease the time needed to check medical records thereby increasing physicians’ bedside availability. Nine resident neurologists participated in a controlled prospective crossover trial over 14 weeks. In the experimental condition, tablets were used in addition to the established medical record. In the control condition, physicians used established systems only. The combined primary outcome measure included changes in total ward round time and relative time shifts between associated work processes. The secondary outcome measure was physicians’ time required to check a medical record vs. physicians’ bedside time. There was a significant main effect on the primary outcome measure (*p* = 0.01). Tablet use accelerated preparing (*p* = 0.004) and post-processing (*p* < 0.001) of ward rounds. Time for conducting ward rounds was unaffected (*p* = 0.19). Checking medical records was faster with tablets (*p* = 0.001) increasing physicians’ bedside time (*p* < 0.001). Tablet use led to significant time savings during preparing and post-processing of ward rounds. It was further associated with time savings during checking medical data and an increase in physicians’ bedside time. Tablets may facilitate clinical data handling and promote workflow.

## Introduction

Recent developments in inpatient care have caused a substantial increase in clinical workload [1]. In their latest annual report from 2012, the German Federal Statistical Office reported a twenty-eight percent increase of cases treated in German hospitals since 1991 [2]. This trend has been paralleled by a twenty-five percent decrease in the number of hospital beds provided, a forty-six percent decrease in length of stay per patient, and a hundred and two percent increase in expenditures [3].

Evidence indicates that the number of diagnoses per patient increases significantly with age [4], meaning that older patients require more intense and costly treatment than their younger counterparts [5]. Whilst this trend is expected to progress in Germany, similar processes have been reported in other countries [6, 7]. It is thus a challenge for health care service providers to efficiently deliver good quality of needed service with fewer resources. Advances in technology offer a considerable potential in this context [8]. In line with this notion, recent research has shown that efficiency and quality of health care delivery can improve through the use of technology [7, 8]. Since clinical practice involves a huge deal of capturing and storing patients’ data, many health care institutions have replaced paper charts with electronic medical records (EMRs) to facilitate data handling [9]. A large body of evidence suggests that EMRs yield both process and structural benefits [10, 11] with several aspects of data management including accessibility and retrieval of patients’ data profiting [12, 13]. Accessing patients’ data can be crucial in making clinical decisions [14], and mobile EMRs grant physicians’ access to patient information whenever they need it [15].

Whilst research has shown that personal digital assistants (PDAs) running an EMR can enhance data handling in clinical settings [13, 16], the hardware has been criticised for limited memory, battery life, and small size [17]. Traditional laptops providing a more common computing experience are considered no suitable alternative due to weight and bulkiness [18]. However, modern tablet computers have ushered in a novel era of mobile computing. These devices have long battery life, considerable storage capacity, larger screens that are convenient for accessing graphical content, and provide true portability. It has thus been pointed out that tablets could potentially fill the gap between PDAs and Laptops [19]. Features that suggest a high suitability of tablets for clinical purposes include ease of disinfection [19] and positive patient perception of tablet use by physicians [20]. Modern tablets further possess a multi-touch operating system for fingers, granting facilitated usability in comparison to their older counterparts.

An early controlled prospective trial comparing paper charts to tablets yielded that preparing ward rounds required significantly less time when tablets were used [21]. In this study, post-processing of ward rounds became redundant, whilst time spent on carrying out ward rounds remained unchanged in the presence of a tablet. However, the results of this research should be treated with great caution; the number of observed ward rounds was small and the study had considerable methodological flaws. A second study investigating the use of tablets running an EMR in an emergency department yielded that physicians’ time spent at the computer workstation decreased significantly due to tablet use [21]. One presumed benefit of such an effect is an increase of doctors’ availability at the bedside. These findings are potentially of great importance considering that the patient satisfaction has been associated with the amount of time patients spend with their physician [22]. However, the amount of time physicians spent at the bedside was not tracked in this study. The authors furthermore point out that their results may have been flawed by inaccurate methods of measurement.

In sum, the above-mentioned research suggest that tablets running an EMR can potentially streamline clinical routine and are highly suitable for the medical environment. Especially interdisciplinary domains, such as neurology, involve a huge deal of data handling and may hence particularly profit from mobile data access. Addressing methodological flaws of earlier studies, a controlled prospective crossover trial investigating the effect of tablets running an EMR specifically designed for tablet use on physicians’ routine is reported. Ward rounds represent a structured daily process amongst other highly dynamic processes in the versatile working environment immanent to hospitals. They hence allow for a systematic evaluation of novel procedures. Based on the literature, our primary hypothesis stated that the use of tablets running an EMR should (1) reduce the overall time required for preparing, conducting and post-processing ward rounds and (2) cause a relative shift from time spent preparing and post-processing towards conducting ward rounds. Our secondary hypothesis was that tablet use should result in a reduction of time physicians require to look up patients’ data. This effect should be paralleled by an increase in time physicians spend at the patients’ bedside.

## Materials and methods

### Participants

Three teams of physicians at the department of Neurology at the Charité University Hospital in Berlin participated in this study. Each team consisted of three resident neurologists resulting in a total of nine participants (2 female, 7 male). Two of the participants were below 30 years of age, five participants were aged between 30 and 39 years, and two were aged between 40 and 49 years. The main sampling criterion was to select physicians who regularly attended ward rounds. All participants met this criterion. Data protection and ethic commission approval were obtained. Prior to data collection, all participants gave informed verbal consent. Strict codes of practice in line with data privacy statements for patient data were applied at any time. Participants were informed about the purpose of the study; however, no information regarding hypotheses was disclosed at this point. IT support was offered throughout data collection. Participants were also instructed that they should not use the tablet if they felt that this would impair delivery of health service in any form (e.g. emergencies). They were free to withdraw from the study at any point. This study was conducted in accordance with *the Helsinki Declaration.*

### Outcome parameters

We sought to employ a single parameter to test our primary hypothesis. This posed a particularly challenging enterprise since the primary hypothesis concerned two distinct dimensions that were calculated from the same data set. These included (1) absolute changes of time required for each individual ward round associated work process (i.e. preparing, conducting and post-processing of ward rounds) and (2) a concurrent relative time change between those work processes. More specifically, the second dimension encompassed shifts from time spent preparing and post-processing ward rounds to ward round conduction. In other words, to test the primary hypothesis, an outcome parameter must be sensitive to absolute time changes per work process and also reflect relative time changes between work processes. Two possible scenarios could be as follows: (1) a major reduction of time required for preparing and post-processing ward rounds paralleled by a minor reduction of time required for ward round conduction would be beneficial in two ways. First, in terms of absolute time savings; second, in terms of a relative shift from time spent at the work station (preparation, post-processing) to time spent carrying out ward rounds. (2) A reduction of time required for preparing and post-processing ward rounds with an inversely proportional increase in time required for ward round conduction would also be beneficial. In the latter scenario, there would be a shift from time spent at the desk towards time spent on the ward in the absence of absolute time savings implicating increased bedside availability. We used data from a pilot study to construct and test a parameter that would be sensitive to changes along both dimensions (hereafter, referred to as efficiency index, abbreviated *I*_eff_). We chose an approach of step-wise adaptations and testing of the *I*_eff_ through Matlab-based computerised simulations (MATLAB^®^ 2008b, The MathWorks, Gatwick, USA) up to the point at which the measure became suitable for investigating our primary hypothesis. Mandatory criteria of the final index were: equal affection by positive and negative changes, potentiation of parallel and weakening of opposing effects along both dimensions, and a linear distribution. The equation for the final definition of the index is given below:

$$I_{\text{eff}} = \frac{{(t_{\text{tot}} + \ln \left[ {\left( {t_{\text{prp}} + t_{\text{pp}} } \right)/t_{\text{prp}} } \right] *t_{\text{tot}} )^{ - 1} }}{100}$$

*t*_wrd_ = ward round conduction time, *t*_prp_ = preparation time, *t*_pp_ = post-processing time, *t*_tot_ = total process time (i.e. *t*_wrd_ + *t*_prp_ + *t*_pp_); ln is the natural logarithm, all times in minutes.

**Fig. 1:**
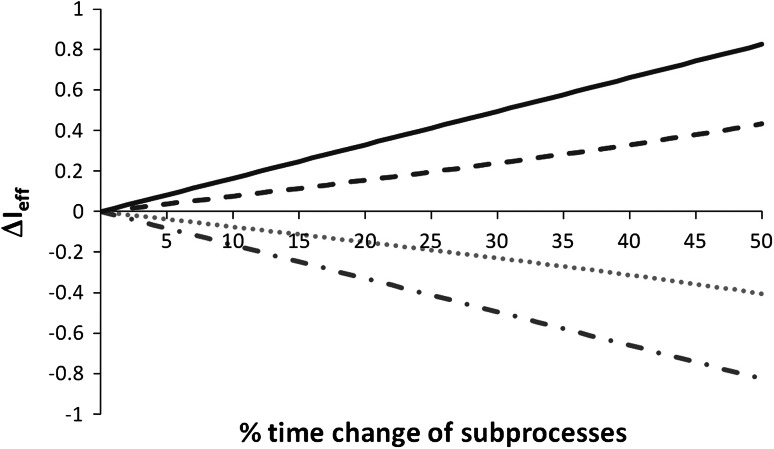
Exemplary simulation of the impact of time changes within work processes on the efficiency index (*I*_eff_; *y*-axis). Rounded mean data from a pilot study were included in this simulation (50, 90 and 30 min for preparing, conducting and post-processing ward rounds, respectively). In the simulation, percentaged changes were applied to each work process individually. Four different scenarios are shown (*x*-axis displays changes in percent): Reduction of all work process times (*solid line*), reduction of preparation and post-processing time with increased ward round conduction time (*dashed line*), increase of all work process times (*dotted line*), increase of preparation and post-processing period and reduction of ward round time (*dotted* and *dashed line*). The simulation shows that the *I*_eff_ reflects both changes in the ratio of preparing, conducting, and post-processing ward rounds as well as time changes within each of these work processes (see “Outcome measures” paragraph in the “Methods” section for details).Combined effects of changes in total time and relative time shifts may reinforce each other if both effects are in the same direction. They may also weaken each other when the direction of effect between the two differs (data not shown for presentational purposes)

In a final simulation, the *I*_eff_ proved to be a valid measure (Fig. 1). Subsequent analyses included the evaluation of time changes of individual work processes (i.e. preparing, conducting and post-processing ward rounds) between the two conditions. The secondary outcome measure was the impact of tablets on physicians’ time spent checking a medical record and physicians’ time spent at the bedside.

### Materials

Physicians used tablet computers (iPad mini^®^, Apple Inc., Cupertino, California, USA) running a mobile electronic medical record (SAP EMR Unwired^®^ Version 1.10, SAP AG, Walldorf, Germany) [23]. Tablet functions were limited and excluded screen shot, camera use, and cloud service use. Users were furthermore not allowed to install applications unless they met data protection requirements as defined by the IT department. EMR features included up-to-date information of ward usage, lab results, functional diagnostics, imaging results, clinical order status, risk factors, demographics, and diagnoses. Whilst the system did not provide the opportunity to enter clinical orders (e.g. imaging or functional diagnostics), clinical tasks and progress notes could be entered and were shared with the back-end system. Vital sign and medication data were not digitally stored and hence paper bound in all conditions. All devices provided access to the internet. For data protection matters, no patient data were saved on tablets at any time; data were instead saved to a back-end that was accessible through the tablet’s front-end. Time records were obtained through the use of wrist watches. Standardised documentation sheets were used to record times. The Institute of Hygiene and Environmental Medicine consented with hygiene standards of the study.

### Data collection

Participants were handed tablets along with a detailed instruction manual. They further received a training session on how to use devices and software. This was done 5 days prior to data collection in the tablet condition to allow physicians to become familiar with the new tool. Precise time records were obtained for the three work processes (preparing, conducting, and post-processing ward rounds) by one of the researchers (JD). The researcher was not a trained physician and accompanied doctors during their ward rounds. He functioned as a passive observer and did not interact with physicians or patients. His duty during ward rounds exclusively consisted in obtaining time records. Physicians that were not escorted by the researcher recorded times for the three work processes through self-monitoring. One time record per team and work process was obtained if ward rounds were conducted by a team of physicians; when physicians conducted ward rounds individually, one time record was obtained per physician and work process. Times were rounded with minutes representing the smallest unit of measurement. Ward round interruptions were recorded and subtracted. In addition to the primary outcome data, the researcher also recorded the times physicians spent at the bedside and the times they used looking up patients’ medical data.

### Design

The study was conducted within groups with one interventional and one control condition. In the interventional condition, a tablet running an EMR specifically designed for mobile use was utilised in addition to the gold standard information systems. The latter consisted of a paper chart and a ward trolley equipped with a laptop running a desktop version of the EMR. We chose to test the hypothesis in a crossover design to reduce the influence of confounding variables. These included individual differences in tablet use and specific patient populations treated by each team (i.e. patients with stroke, inflammatory and autoimmune disease, peripheral nervous system disorders etc.).

**Fig. 2:**
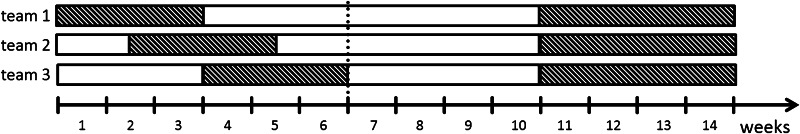
Figure displays the study design. *Horizontal bars* represent the three teams of physicians that participated in the study. *Shaded areas* indicate periods in the interventional condition. The *dashed vertical line* at week six displays the date for scheduled rotation of physicians between teams and hence marks the transition between phase I and phase II of the study (see “Methods” section for details). Teams participated in both conditions. Note that the randomised introduction period (phase I) was followed by a subsequent classic block design (phase II)

Accounting for scheduled absence (e.g. holiday, on-call-duties), a power analysis revealed that the study period needed to comprise 14 weeks. This was particularly important since several aspects that could not be accounted for prior to data collection posed the risk to limit the number of available data points. Amongst others, these included (1) unavailability due to sickness or emergencies and (2) ward rounds carried out by a team of physicians and not individually (i.e. ward rounds with a team of three physicians providing one data point per work process; three ward rounds each carried out by physicians individually providing three data points per work process). We had to, moreover, divide the investigation period into two phases since physicians were scheduled to be reallocated between teams 6 weeks after data collection initiation which conflicted with the crossover design. The 14-week study period was hence split into a 6 week (phase I) and an 8 week (phase II) period. We chose a time-shifted introduction of the tablet in phase I to control for a possible sequence effect of tablet use on subsequent performance in the control condition. The order of teams was randomised. Phase II was conducted in a classic block design (Fig. 2).

### Data processing and statistics

SPSS^®^ Statistics (Version 19, IBM Corporation, Armonk, USA) was used to run Analysis of Covariance (ANCOVA), *t* tests, and bootstrapping procedure. Power analysis, primary outcome measure calculation, and outlier corrections were performed in Matlab (MATLAB^®^ 2008b, The MathWorks, Gatwick, USA). Data points above or below twice the interquartile range from the mean were considered outliers and excluded from data analysis. An ANCOVA was performed to test for between-group differences regarding the primary outcome measure. The statistical model included covariates as potentially confounding variables including number of patients seen during ward round, type of ward round (i.e. team or individual ward round), and team membership. Only complete data sets including time records for all associated work processes were used for *I*_eff_ analysis. ANCOVAs were also run to individually analyse the effects of each of the work process on the *I*_eff_. Tukey–Kramer procedure was used as multiple comparison alpha-error accumulation correction method. Secondary outcome (time required looking up medical data and bedside time) measures were tested between groups using a two-tailed paired *t* test. Group data are reported as mean ± standard deviation throughout. Two methods of data collection were applied in this study (passive observation vs. self-monitoring) Subsequent to data collection, 95 % confidence intervals (95 % CI) of variation coefficients (CV) for time records of all three work processes per team were estimated by bootstrapping with 1,000 replications. Non-overlap of intervals indicated that data reliability did not differ significantly between methods of data collection (data reported as mean CV with the lower limit and upper limit of its 95 % CI in parentheses) [24].

## Results

### Times for preparing, carrying out, and post-processing ward rounds

**Fig. 3:**
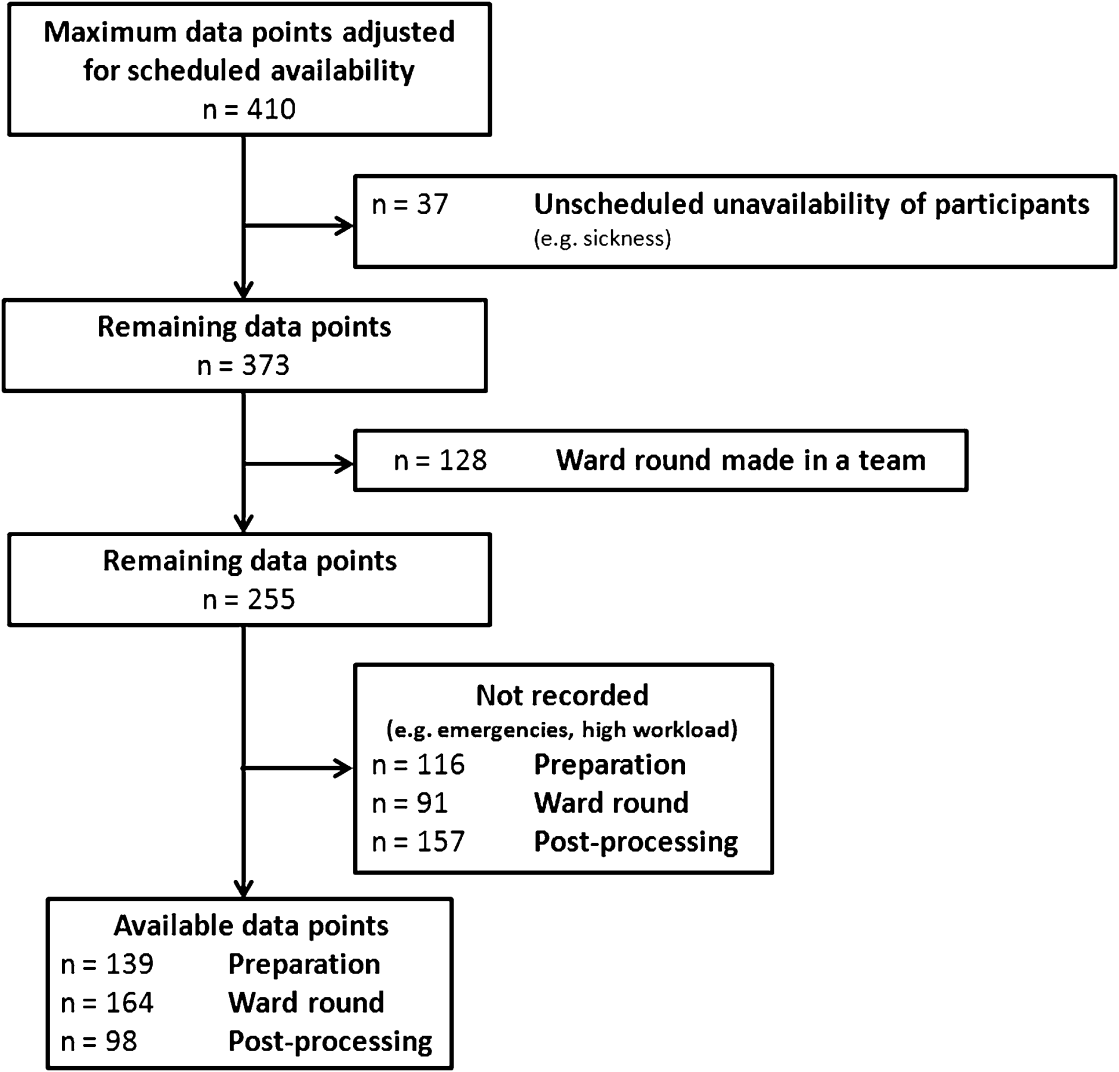
Flowchart displaying the make-up of data points at the end of data collection. Most significant causes of dropout were due to ward rounds made in a team and lack of time documentation

All physicians participated throughout the entire study period. Times for a total of 164 ward rounds were recorded (72 with tablet and 92 without tablet). Preparation times were measured for 139 ward rounds (52 with tablet and 87 without tablet). Post-processing times were recorded for 98 ward rounds (42 with tablet and 56 without tablet). A detailed description of data composition is given in Fig. 3. A considerable amount of data points was collected by one of the researchers (preparing ward rounds: 72 data points, carrying out ward rounds: 75 data points, post-processing ward rounds: 58 data points). The remaining data points were collected by physicians through self-monitoring. Obtained data points did not differ significantly with respect to 95 % confidence intervals of variation coefficients (CV) between teams regarding preparing [team 1: 0.55 (0.45–0.62), team 2: 0.44 (0.34–0.51), team 3: 0.54 (0.43–0.63)], conducting [team 1: 0.64 (0.47–0.75), team 2: 0.48 (0.38–0.54), team 3: 0.45 (0.38–0.49)] and post-processing (team 1: 0.89 (0.54–1.07), team 2: 0.64 (0.45–0.78), team 3: 0.62 (0.46–0.74)) ward rounds.

**Fig. 4:**
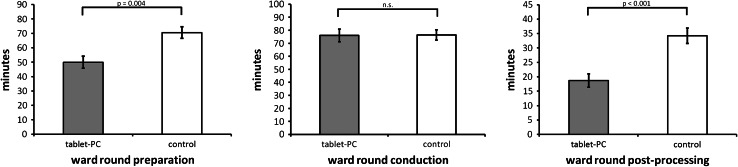
Mean values of time required to carry out ward round work processes in minutes for interventional and control conditions. The time required for preparing and post-processing ward rounds decreased significantly through tablet use. No effects of tablet use on time required for carrying out ward rounds were observed. *Errors bars* indicate the standard error of the mean

An ANCOVA with type of ward round, number of patients visited during ward round, and team membership as covariates was performed to examine the effects of tablet use on the primary outcome measure. The analysis revealed that the efficiency index (*I*_eff_) was significantly higher for the interventional condition as compared to the control condition (−1.47 ± 0.72 vs. −2.03 ± 0.88; *F*(1,94) = 6.94, *p* = 0.01). ANCOVAs further revealed that there was a significant main effect of tablet use on time required for preparing (interventional condition: 49.9 ± 30.3 min, control condition: 70.5 ± 36.9 min; *F*(1,137) = 8.46, *p* = 0.004) and post-processing (interventional condition: 18.7 ± 14.9 min, control condition: 34.2 ± 19.7 min; *F*(1,96) = 14.3, *p* < 0.001) ward rounds. Times for ward round conduction remained unchanged (interventional condition: 76.0 ± 41.0 min, control condition: 76.0 ± 38.2 min; F(1,162) = 1,77, *p* = 0.19) (Fig. 4).

### Time spent at the bedside

Physicians’ time spent looking up a medical record during ward rounds was recorded for 269 cases in the interventional condition and for 264 cases in the control condition. For the experimental condition, nine data points were identified as outliers and removed from the data set. For the control condition, four outliers were removed. Results indicated that looking up medical data was significantly faster per patient with tablets than without tablets (4.6 ± 2.5 min vs. 5.4 ± 2.7 min per patient; *t*(259) = −3.46, *p* = 0.01).

**Fig. 5:**
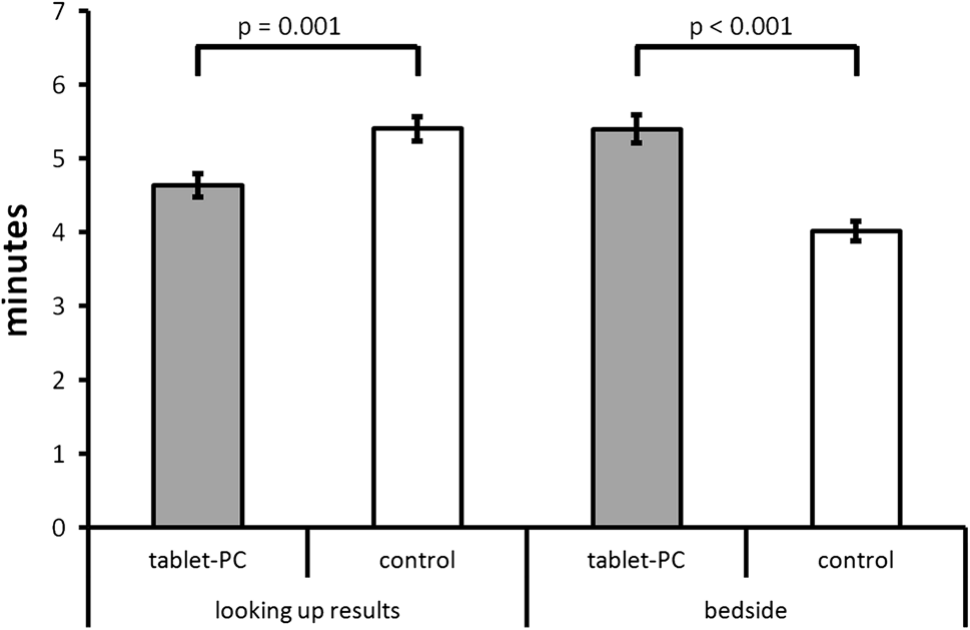
Mean values for time required to carry out work processes associated with ward rounds. Looking up medical records required significantly less time when tablets were used. Tablet use was also associated with a significant increase in physicians’ time spent at the bedside. *Errors bars* indicate the standard error of the mean

Physician’s time spent at the bedside during ward rounds was measured for 283 cases in the interventional condition and for 284 cases in the control condition. For the interventional condition, fourteen data points were identified as outliers and removed from the data set. For the control condition, fifteen outliers were removed. Results indicated that physicians spent significantly more time with each patient at the bedside when using a tablet (5.4 ± 3.1 min vs. 4.0 ± 2.4 min; *t*(268) = 6.13, *p* < 0.01) (Fig. 5).

## Discussion

To our best knowledge, this is the first controlled prospective trial investigating quantitative effects of tablets running an EMR on clinical routine in an inpatient setting. Results indicate that tablet use substantially reduced physicians’ time spent on preparing and post-processing ward rounds, while the duration for conducting ward rounds remained unchanged. Tablet use also reduced the amount of time physicians required to look up medical records which was associated with an increase in physicians’ time spent at the bedside.

### Increased efficiency during ward round work processes

The findings add on to the body of evidence indicating that information technology can enhance efficiency within clinical settings [8, 11]. Most of the existing literature investigated the impact of tablets compared to paper record or desktop computer use in emergency [14, 25] and ambulatory care [26]. These studies mainly evaluated qualitative outcome parameters in clinical routine. Results indicated increased productivity [17], improved patient–physician interaction and workflow [27], enhanced data access [16], and possibly optimised patient outcome [13]. However, only two studies employed objective quantitative measurements allowing for unbiased comparison and cost-effectiveness analyses [21, 28]. Only one of these studies compared the impact of a tablet on ward round performance. Fränkel reported a decrease in physicians’ time spent on preparing ward rounds, whilst the time needed for ward round conduction remained unchanged [21]. In this study, post-processing of ward rounds became redundant since clinical documentation was fully completed during ward rounds. This contrasts our findings of decreased post-processing times. However, results by Fränkel were weakened by a small sample size of only ten ward rounds per condition, whilst details on data collection and statistical analysis were not reported clearly. Furthermore, in contrast to our setting, times were recorded during ward rounds of a consultation service and not residents of the ward. Results of this study should, hence, be treated with great caution.

The present study provides novel evidence indicating that the use of tablets can enhance efficiency of ward round performance. In addition to a reduced total process time, results show a relative shift from preparing and post-processing towards ward round conduction and an increase in time spent at the bedside. It is not entirely clear why tablet use accelerated data retrieval in our study. However, the mobility of data access offers an appealing explanation. Looking up patient data at the workstation becomes largely redundant when tablets provide mobile access to these data [13, 15, 19]. It is also possible that the tablet-specific user interface facilitated data access and hence led to faster data retrieval.

The finding that tablet use did not affect the length of time required for conducting ward rounds deserves close attention. Existing literature suggests that facilitated access to patient records and related information (e.g. drug database, internet search) may lead to an increase in time required for ward round conduction [16]. The present study provides clear evidence that this is due to a shift from time spent looking up patient data towards time spent at the bedside (see paragraph below). Since a detailed documentation of procedures during ward rounds was beyond the scope of the present investigation, it remains unclear if and how tablet use impacted on single procedures. Further investigations are hence necessary. Results could provide a rationale to further advance mobile EMR software.

### Promotion of bedside time

Consistent with previous research, a highly significant finding from the secondary outcome measure clearly indicated that checking a medical record required less time when using a tablet [21]. The study conducted by Horng et al. lacked a mechanism to investigate if time savings for looking up patient data led to increased time at the bedside. In our study, however, a significant increase in time spent at the bedside was observed. This is unsurprising considering that physicians perceive it as useful to employ tablets for discussing clinical evidence such as lab results and radiology films with patients [19, 27]. An increase in physicians’ time spent at the bedside is furthermore likely to increase patient satisfaction [22]. It remains unclear whether an increased availability of physicians at the bedside necessarily improves patient outcome [17, 28]. Future research should aim to shed light on this issue. Our findings underline the workflow-enhancing potential of tablets as outlined in the literature. However, it is not yet entirely clear why tablets as additional device outpace the established medical records when looking up patient data. Further investigation will be required to better understand the underlying mechanisms.

### Usability

Health and safety matters are a substantial concern in the clinical environment. Whilst disinfection of tablet computers proved to be quick and simple, fast session lockout time posed a considerable shortcoming. To grant secure data handling, key lock was automatically activated on devices after 5 min. Reactivation of keys required a new log-in by entering a six digits alphanumeric code. Software sessions timed out after 2 min and also required the user to enter a password to re-access patient data. In combination, the log-in processes were time consuming and led to delays. Data security, however, is a major concern regarding the introduction of mobile devices [15, 17, 18]. Fingerprint lock appears a suitable alternative that could possibly avoid time loss.

The most evident technical issue consisted in prolonged load times of medical data due to varying network coverage. This problem occurred in particular when retrieving large files. In rare cases, patient data were only available through the workstation and could not be accessed through the tablet. We consider this issue to be of temporary nature considering the rapid advance of network infrastructure and technology.

### Limitations

Despite addressing methodological flaws of earlier studies such as small sample size and a retrospective design, there were some potential technical and methodological limitations to this study.

It cannot be ruled out that occasionally long load times impeded physicians’ confidence in the new device or even caused them to resign checking medical records in exceptional cases. Supporting this view, previous research has shown that full and reliable functionality are essential to avoid losing possible benefits of tablets for clinical routine due to nonuse and refusal of newly introduced systems [18]. Fast and complete data access [15], full functionality compared to desktop workstations [19], and legibility as well as data presentation are key in this context [15]. These factors should be accounted for in the context of future studies and clinical use of tablets.

Furthermore, a considerable amount of data was collected by one of the researchers and was, therefore, possibly subject to bias. However, coefficients of variation indicated that reliability of data was not influenced by method of collection. Reliable data collection across teams can, hence, be inferred. Data dropout is another possible concern. However, it is practically not possible to control for contributing factors such as emergencies, workload, and division of duties influencing ward round conduction and data collection.

### Conclusion and perspective

This is the first controlled prospective trial providing evidence in support of the hypothesis that the use of a mobile EMR can enhance the efficiency of clinical routine in an inpatient setting. Results from this study suggest that adding mobility to medical information technology can help to provide more efficient and patient-oriented health care delivery in the face of increasing demands. Although this study was conducted in a neurological setting using an EMR that met specific requirements, it is likely that health care professionals from other disciplines may also benefit from mobile data access. Future research should aim to shed light on this question. Future studies should furthermore aim to examine the causes for the time savings that were observed in this study. Such a research design would track information retrieval by physicians, ideally through automatic electronic data capturing. Further parameters that deserve close attention include patient-oriented outcomes such as patient satisfaction.
